# SNAPIN Regulates Cell Cycle Progression to Promote Pancreatic β Cell Growth

**DOI:** 10.3389/fendo.2021.624309

**Published:** 2021-06-14

**Authors:** Mengxue Jiang, Zhijian Kuang, Yaohui He, Yin Cao, Tingyan Yu, Jidong Cheng, Wen Liu, Wei Wang

**Affiliations:** ^1^ Department of Endocrinology, Xiang’an Hospital of Xiamen University, School of Medicine, Xiamen University, Xiamen, China; ^2^ Fujian Provincial KeyLaboratory of Innovative Drug Target Research, School of Pharmaceutical Sciences, Xiamen University, Xiamen, China

**Keywords:** SNAPIN, β cells, proliferation, cell cycle, diabetes

## Abstract

In diabetes mellitus, death of β cell in the pancreas occurs throughout the development of the disease, with loss of insulin production. The maintenance of β cell number is essential to maintaining normoglycemia. SNAPIN has been found to regulate insulin secretion, but whether it induces β cell proliferation remains to be elucidated. This study aimed to explore the physiological roles of SNAPIN in β cell proliferation. SNAPIN expression increases with the age of mice and SNAPIN is down-regulated in diabetes. KEGG pathway and GO analysis showed that SNAPIN- interacting proteins were enriched in cell cycle regulation. B cell cycle was arrested in the S phase, and cell proliferation was inhibited after SNAPIN knockdown. The expression of CDK2, CDK4 and CCND1 proteins in the S phase of the cell cycle were reduced after SNAPIN knockdown, whereas they were increased after overexpression of SNAPIN. In addition, insulin protein and mRNA levels also increased or decreased after SNAPIN knockdown or overexpression, respectively. Conclusions: Our data indicate that SNAPIN mediates β cells proliferation and insulin secretion, and provide evidences that SNAPIN might be a pharmacotherapeutic target for diabetes mellitus.

## Introduction

Diabetes is a prevalent disease worldwide, and it has become a serious social health problem (1). It ultimately results in a deficiency of functional pancreatic β cells (2).The maintenance of β-cell number and islet mass is essential for maintaining normoglycemia (3, 4). Human β cell replication rates are very low, and the cells are only capable of replication for a brief period after birth (5).

Type 1 diabetes mellitus (T1DM) is associated with impaired β cell mass (6).The pathogenesis of type 2 diabetes is more variable, and it consists of insulin resistance and defective insulin secretion (6). More specifically, type 2 diabetes mellitus (T2DM) is associated with genetic predisposition and environmental factors, such as obesity and diet (7). During pregnancy and the early stages of diabetes, compensatory proliferation of pancreatic β cells occurs in response to changes in blood glucose (8, 9). Glucose itself is able to stimulate β cell replication (10), and several lines of evidence indicate that the terminally differentiated pancreatic β cells retain significantly proliferative capacity *in vivo* (11, 12). This proliferative capacity has attracted considerable research attention in terms of developing therapeutic strategies for diabetes mellitus. Although a number of studies concerning differentiated β-like cells from embryonic stem cells or induced pluripotent (adult) stem cells are in progress, the low conversion efficiency of β cells from stem cells remains a challenge for developing cell-based therapies (13). Glucokinase signaling, carbohydrate response element-binding protein (ChREBP), nuclear factor of activated T-cells (NFAT), platelet-derived growth factor (PDGF), CDK4/6 and TCF7L2 have all been reported to stimulate human β cells proliferation (14–19). Therefore, the mechanisms regulating β cell mass have been revealed that underlie the development of T1DM and T2DM, which is important for developing novel therapeutic approaches for diabetes. This proliferative capacity has attracted considerable research attention in terms of developing therapeutic strategies for diabetes mellitus.

SNAPIN is a protein that interacts with SNARE complexes during synaptic transmission and was first reported by Jeffrey M. Ilardi in 1999 and it was first identified in neurons and located on synaptic vesicle Membranes (20). It is also a component member of the BLOC-1 complex and BORC complex (21). The BLOC-1 complex is required for normal biogenesis of lysosome-related organelles (LRO), such as platelet-dense granules and melanosomes (22), and the BORC is required for lysosome positioning in mammalian cells (21). Increasing evidence shows that SNAPIN is important for retrograde axonal transport (23), late endosomal-lysosomal trafficking (24), and glucose-induced insulin exocytosis (25). It is also believed to be involved in a variety of signal transduction and intracapsular transport/fusion functions (26).

SNAPIN is specifically expressed in the endocrine department of the pancreas. Diffuse cytoplasmic staining has been observed, and the cells were clustered into punctate structures, which co-located with insulin-secreting granules (27).The insulin secretion may be caused by the interaction between the c-terminal H2 region of SNAPIN and sn-1 region of snap-25 in the SNARE complex (27, 28), which initiates the process of insulin secretion particle targeting, tethering, initiation and membrane fusion (27, 29). These exocytosis processes are mediated by the Munc18/SNARE complex (30).

In addition, SNAPIN is a target of protein kinase A (PKA) (31), which is a critical regulator of glucose-stimulated insulin exocytosis in pancreatic β cells by promoting the interaction and assembly of insulin secretory vesicle-associated proteins Snap25 and TMEM27 (32). SNAPIN is significantly correlated with the TMEM27 gene, which codes a membrane protein cleaved and shed by pancreatic beta cells that have been proposed as a beta cell mass biomarker (33). This indicates that SNAPIN may also be a biomarker for beta cells. The function of SNAPIN in β cell growth is poorly understood, and our findings reveal that the overexpression of SNAPIN in Min6 cells can promote cell proliferation and is promising in achieving the goals of regenerative medicine for diabetes treatment.

## Materials and Methods

### Cloning Procedures

Snapin full length was PCR-amplified from cDNAs and cloned at XhoI and BamHI sites of PCDH-3xFlag-3xHA-EF1-puromycin vector. Primers were designed using the Primer Premier 5.0 software (Premier Biosoft International, Palo Alto, CA).

### Lentivirus Packaging and Infection

HEK293T cells were seeded in culture plates for 12 hrs and transfected with lentiviral vectors together with packaging vectors, pMDL, VSVG, and REV, at a ratio of 10:5:3:2 using Lipofectamine 2000 for 48 hrs. Virus was collected, filtered and added to β cells in the presence of 10 μg/mL polybrene (Sigma, H9268), followed by centrifugation for 30 mins at 1,500 g at 37°C. Medium was replaced 12 hrs later. The transduction efficiency was evaluated by immunoblotting analysis.

### shRNA Infection

The packaging process of lentiviruses particles was the same as above. Primers sequences were in the **Table 1**. The transient transfection of plasmids into the cells was performed by using Lipofactamine 2000 reagents (Invitrogen) according to the manufacturer’s instructions.

**Table 1:** shRNA targeting sequencing and primers for SNAPIN cloning.

Gene	Forward (*5’→ 3’*)	Reverse (*5’→ 3’*)
shRNA-1	CCGGCAACCTAGCTACAGAACTGTGCTC	AATTCAAAAACAACCTAGCTACAGAACTG
(plko.1)	GAGCACAGTTCTGTAGCTAGGTTGTTTTTG	TGCTCGAGCACAGTTCTGTAGCTAGGTTG
shRNA-2	CCGGGAACAAATTGACAACCTAGCTCTC	AATTCAAAAAAGAACAAATTGACAACCTA
(plko.1)	GAGAGCTAGGTTGTCAATTTGTTCTTTTTG	GCTCTCGAGAGCTAGGTTGTCAATTTGTTC
shRNA-1	TCGACAACCTAGCTACAGAACTGTGTTCAAG	GATC GAAAAAACAACCTAGCTACAGAACT
(psicor)	AGACACAGTTCTGTAGCTAGGTTGTTTTTTC	GTGTCTCTTGAACACAGTTCTGTAGCTAGGTTG
shRNA-2	TCGAGAACAAATTGACAACCTAGCT TTCAA	GATC GAAAAAAGAACAAATTGACAACCTA
(psicor)	GAGAAGCTAGGTTGTCAATTTGTTC TTTTTTC	GCTTCTCTTGAAAGCTAGGTTGTCAATTTGTTC
SNAPIN	AGAGAATTCGGATCCATGGCCGCGGCTGGTT	CTTCCATGGCTCGAGTTTGCTTGGAGAACCAGG
(PCDH)		

### Cell Lines

The mouse pancreatic Min6, the rat pancreatic INS1 and the HEK-293T cell lines were obtained from ATCC. Min6 cells were maintained in high-glucose DMEM medium(BI) supplemented with 15% v/v fetal bovine serum (FBS, BI), 50 mmol/L 2-mercaptoethanol, and 1% penicillin-streptomycin at 37°C and 5% CO2 in a humidified atmosphere in an incubator. HEK-293T was cultured in DMEM medium supplemented with 10% v/v fetal bovine serum (FBS, BI) and 1% penicillin-streptomycin in an incubator at 37°C and 5% CO2 in a humidified atmosphere in an incubator. The rat pancreatic INS1 cells were cultured in RPMI1640 (Hyclone) containing 10% v/v fetal bovine serum (FBS, BI), 1% penicillin-streptomycin 1% glutamine, 1 mM sodium pyruvate, and 50 μM β-mercaptoethanol (sigma).

### Animal

Male KM mice weighing approximately 18–25 g were purchased from the Animal Center of Xiamen University. Mice were humanely housed at 22 ± 2°C with 12-h light/dark cycles. All animals had free access to food and water. All animal studies were approved by the ethical review committee of Xiamen University and followed the regulations of the National Institutes of Health guidelines on the care and welfare of laboratory animals. Db/Db and HFD mice were obtained from Li Ming Yu lab at Xiamen University.

### Immunohistochemistry

SNAPIN and insulin were analyzed by immunohistochemical staining of frozen sections from adult mouse pancreas fixed O/N in 4% PFA in PBS at RT, pH 7.4, cryoprotected in 30% sucrose in PBS, embedded in Tissue-Tek (Sakura; Værlose, Denmark), and cut into 9-μm sections on a Leica cryostat. Antigen retrieval was performed by microwave treatment for 4 mins at 600 W in 200 mL 0.01 M citrate buffer, followed by 15 mins at 250 W, and finally left to cool for 20 mins. Tissue sections were rinsed in TBS, quenched in 3% H2O2 for 5 mins, and rinsed again. Then, 10% goat serum was used for blocking for 1 hr before overnight incubation with the purified rabbit anti-SNAPIN antibodies diluted 1:200 and rabbit anti-insulin antibodies diluted 1:400 in 10% goat serum blocking buffer. Sections were washed three times for 5 mins each in TBS between incubations with primary antibody and the following incubations with biotinylated secondary antibody (Zymed; Aarhus, Denmark) for 30 mins. Bound antibodies were visualized with the DAB after a 3 mins incubation period and a subsequent wash in TBS, and the slides were counterstained with hematoxylin. Images were collected with an Olympus (Tokyo, Japan) BX51 microscope and captured using a chilled Hamamatsu C5810 CCD camera (Hamamatsu City, Japan) and Image Pro Plus 4.5 software.

### Nuclear Extraction of Cells

SNAPIN was overexpressed in Min6 cells. All steps were performed on ice with ice-cold reagents in pre-centrifuge tubes. We first used ice-cold 1xPBS to wash cells and scrape cells gently into a 15mL tube. They were centrifuged for 5 mins at 500 rpm, 4°C. Then the pellet was resuspended in 1 mL ice cold BUFFER1 (1 M HEPES, PH 8.0, 1 M Mgcl2, 1 M KCl and 1 M DTT) and incubated for 15 mins on ice to allow cells to swell. Np40 was added to vortex for 10 S. Cells were centrifuged for 3 mins at maximum speed, then supernatant was aspirated with pipet, which was the cytoplasmic fraction. The pellet was resuspended in cold BUFFER2 (1 M HEPES, PH 8.0, 1 M Mgcl2, Glycerol, 1 M Nacl, 0.5M EDTA and 1 M DTT), vortexed 30 S and rotated vigorously at 4°C for 30 mins. The pellet was centrifuged for 15 mins at maximum speed, and the supernatant was removed and transferred to a fresh, chilled tube. Assay for protein concentration for immunoblotting analysis.

### Confocal Immunofluorescence Microscopy

β cells were fixed in 4% paraformaldehyde for half an hour at room temperature. Then, cells were washed three times with PBS for 5 mins at room temperature. 0.1% Triton-100 was added for permeation for 10 mins, and cells were incubated in a blocking buffer (5.0% BSA in PBS) for 1 hr at room temperature. Then we performed immunostaining using the primary antibodies SNAPIN (1:50) and insulin (1:800) incubated overnight at 4°C, followed by fluorophore-conjugated secondary antibodies. The immunolabeled cells were analyzed with a Carl Zeiss LSM5 EXITER or Leica TCS SP8 STED laser scanning confocal microscope. The green channel was imaged using a 488-nm laser line (120 mW/cm2, 2.5%). The red channel was imaged using a 559-nm laser (120 mW/cm2, 2.0%). Images were acquired in 4-s intervals (frame time 3.9 s). A pulsed 375-nm laser (10 MHz) was applied for uncaging experiments in the entire field of view for eight frames (3.2 s/frame). The dual scanner setup allowed for simultaneous laser stimulation and confocal imaging. This permitted the acquisition of cellular responses that occurred during or immediately after laser stimulation.

### Cell Cycle and Flow Cytometry

Min6 cells that underwent SNAPIN overexpression or knockdown were added to 1 mL cold PBS to re-suspend cell pellet and vortexed gently and an equal volume of cold absolute ethanol (store at -20°C) was added dropwisely to cell suspension in the same 15 mL tube. Then, it was stored at – 20°C for at least 2 hrs. Before staining, cells were washed twice with cold PBS and centrifuged at 4°C, 3000g, for 5mins, and supernatant was completely removed. PI staining solution was added, and it was incubated at 37°C for 15 mins. Data were acquired on a flow cytometry. Then, we used PI to stain Min6 cells that transfected shRNA for 15 mins. Cells or nuclei were filtered through 40-mm sieves, and flow cytometry was performed on a 4-laser, LSRII (BD Biosciences). Data were analyzed with FlowJo software.

### Cell Proliferation Assay

Min6 cells that underwent SNAPIN overexpression or knockdown were seeded into a 96-well plate and were incubated with the medium for 24 hrs. The cell viability was measured by MTS analysis using CellTiter 96 AQueous One Solution Cell Proliferation Assay (Promega). For the measurement of cell viability, 500μL of medium/MTS solution mix (20μL MTS/mL medium) was added per well. The plate was incubated at 37°C for 1 hr. Then, the absorbance at 490nm was measured using an automatic microplate reader. Cell viability was measured every 24 hrs for consecutive four days.

### Ethynyldeoxyuridine (EdU) Analysis

Proliferating cells were assessed using a Click-iT™ EdU Proliferation Assay (Invitrogen) following the manufacturer's protocol. Briefly, Min6 cells that underwent knockdown were inoculated seeded into a 96-well plate (5000 cells/well). Then, 10 um EdU labeling media was added and cells were incubated for 24hrs. 50 µL Click-iT™ EdU fixative was added to each well for 5 mins and 50 µL 1X Click-iT™ reaction cocktail to each well for 30 mins. Then, 1.5% BSA was added to block cells for 5 mins. After washing several times, 100 µL Amplex™ UltraRed reaction mixture was added for 15 mins and followed by incubated with 10 µL Amplex™ UltraRed stop reagent. Fluorescence was measured with excitation maximum of 568 nm and emission maximum of 585 nm.

### Annexin V-FITC/Propidium Iodide (PI) Staining

Apoptosis of β cells were measured by Annexin V/FITC(BD Bioscience)according to the manufacturer’s protocol. Cells were harvested and resuspended in 100 μL binding buffer, followed by staining with 5 μL FITC− conjugated Annexin V and 5 μL PI in the dark for 15 mins at room temperature. Then, 00 μL binding buffer was added to resuspend cells. Cells were detected by flow cytometry (BD Biosciences). In the early stage of apoptosis, cell membranes were stained with FITC− conjugated Annexin V, whereas nuclei were not stained with PI. In the late stage of apoptosis, cells were stained with both FITC− conjugated Annexin V and PI. The results were analyzed by FlowJo vX.0.7 (FlowJo LLC).

### Insulin Secretion in bb Cells

In order to test the secretory response to glucose, Min6 cells were cultured on glass coverslips for 24 hrs. After 24 hrs of plating, Min6 cells were incubated for 1 hr at 37°C in KIRH BUFFER (136 mmol/L NaCl, 4.7 mmol/L potassium chloride (KCl), 1.2 mmol/L KH2PO4, 1.2 mmol/L MgSO4, 5 mmol/L NaHCO3, 1 mmol/L CaCl2, 10 mmol/L HEPES, and 0.5% BSA, pH 7.4) supplemented with either 2.8 mmol/L or 16.7 mmol/L glucose. Insulin was detected by ELISA (wide range mouse insulin immunoassay kit, catalogue number: 32100). 5 µL of standard and sample and 100 µL detection antibody was added to each well followed by incubation at room temperature for 90 mins. After washing for several times, 100 µL of substrate solution was added to each well at room temperature for 15 mins followed by adding 100 µL of stop solution and detecting at 450 nm immediately. Cells were fixed with 4% paraformaldehyde for 20 mins, and fixed cells were permeabilized with 0.5% Triton-100 for 15 mins, treated with 0.1% Tween 20 in phosphate buffered saline for 5 mins and blocked with 5% FBS for 40 mins before incubation with the primary antibody against SNAPIN (1:50; proteintech) and insulin (1:800; Cell Signaling Technology) overnight at 4°C, followed by incubation with rhodamine-labeled anti-mouse IgG secondary antibody (1:200; Chemicon, Temecula, USA) for 1 hr at room temperature. Nuclear localization was counterstained with DAPI.

### Tissues and Cells RNA Isolation

Mouse tissues were isolated from 5 days, 2, 3, 4 and 8 weeks mice. Total RNA was then extracted using TRIzol reagent (Gibco-BRL) and following the manufacturer’s instructions.

### Real-Time Reverse Transcriptase-Polymerase Chain Reaction

Briefly, total RNA was extracted using the TRIzol reagent (Invitrogen) and complementary DNA was generated using the PrimeScript™ reverse transcript reagent Kit (Takara, Tokyo, Japan). From 1 μg RNA aliquots, we synthesized cDNA using random hexamers or oligo (dT), and reverse transcriptase, following the manufacturer’s protocol. The reactions were run for 40 cycles at 30°C for 10 mins, 42°C for 20 mins, 99°C for 5 mins, and 4°C for 5 mins using real-time PCR detection system (Bio-Rad, Hercules, USA) according to the manufacturer’s instruction and verified on gel. Real-time reverse transcriptase-polymerase chain reactions (real-time RT-PCRs) were performed using the Power SYBR Green PCR Master Mix (Roche) and a 7500 Real-Time PCR Detection System (Applied Biosystems). Actin was used as an internal control. Primer sequences are listed in **Table 2**. All experiments were performed in triplicate, and the data are shown as mean ± SD.

**Table 2:** Real-time PCR primer sequences.

Gene	Forward (*5’→ 3’*)	Reverse (*5’→ 3’*)
SNAPIN	AGCTACAGAACTGTGCCGGATCAA	GCTTGGAGAACCAGGAGGGTAAA
Insulin1	AGGACCCACAAGTGGAACAA	GCTGGTAGAGGGAGCAGATG
Insulin2	GGACCCACAAGTGGAACAAC	GTGCAGCACTGATCCACAAT
CDK2	AATGCAGAGGGGTCCATCAA	GGAGTAGTACTTGCAGCCCA
Actin	TGTTACCAACTGGGACGACA	GGGGTGTTGAAGGTCTCAAA

### Western Blotting

Cells were seeded at the appropriate density, and total protein of cells or tissues were using cell lysis buffer containing (50 mM Tris-HCl (pH 7.4), 150 mM NaCl, 1 mM EDTA and 1% Triton X-100)containing protease inhibitor cocktail (sigma, P2714-1BTL) on ice for 30 mins followed by centrifugation. Protein concentrations was determined with a Bicinchoninic Acid Assay Kit (Beyotime Biotechnology). Protein (20 μg/lane) was separated by 10–15% sodium dodecyl sulfate‐polyacrylamide gel electrophoresis (SDS-PAGE) and transferred onto polyvinylidene fluoride membranes (Millipore, Billerica, MA). The membranes were blocked for 1.5 hrs in Tris‐buffered saline and Tween 20 (TBST, pH 7.6) containing 5% non‐fat milk powder at room temperature and incubated with primary antibodies at 4°C overnight. The membranes were then washed three times with TBST for 15 mins each and incubated with anti‐rabbit secondary antibodies (1:5,000 in TBST) conjugated to HRP for 1 hr at room temperature. The blots were then developed in the dark by using an ECL detection kit (Proteintech). Band intensities were quantified by ImageJ 1.45 software (NIH, USA). Antibodies are listed in **Table 3**.

**Table 3:** Antibodies used in Western blotting, immunohistochemistry and immunofluorescence.

Antibody	Dilution	Host Species	Supplier
β-Actin	1:1000	Rabbit	proteintech
Snapin	1:1000	Rabbit	proteintech
Snapin	1:1000	Rabbit	Synaptic System
Insulin	1:800	mouse	Cell Signaling
Insulin	1:400	Rabbit	proteintech
Flag	1:1000	mouse	Sigma
HA	1:1000	Rabbit	Santa Cruz
CDK2	1:1000	Rabbit	proteintech
CDK4	1:1000	Rabbit	proteintech
CCND1	1:1000	Rabbit	proteintech
PARP	1:1000	Rabbit	Cell Signaling
HSP60	1:1000	Rabbit	proteintech
BLOC1S1	1:1000	Rabbit	ABclonal
HRP-anti-rabbit IgG	1:1000	Rabbit	Beyotime
CY3-anti-rabbit lgG	1:200	Rabbit	Beyotime
CY3-anti-mouse lgG	1:200	mouse	Beyotime
FITC-anti-mouse lgG	1:200	mouse	Beyotime

### Immunoprecipitation Assay

Two days after transfection, the cells were lysed in lysis buffer (1% Triton X-100, 50 mM Tris–HCl (pH 7.4), 150 mM NaCl, 1 mM EDTA, 1% sodium deoxychlate) with protease inhibitor cocktails. Pre-washed Flag-beads were added. After immunoprecipitation, Flag-Beads were washed 4-5 times with lysis buffer (containing protease inhibitor). Flag peptides were added to elute Flag-tagged proteins. 4xSDS loading buffer was added into the supernatant after enrichment and the sample was boiled in the metal bath for 10min before being The elutes were then subjected to SDS–PAGE and Western blotting analysis.

### LC–MS Analysis and Database Search

Each immunoprecipitation (IP) was carried out in triplicate. Min6 cells expressing Flag-SNAPIN or PCDH vector were grown on 15 cm plates until 90% confluent and then subjected to IP. The elutes after IP were firstly reduced in 20 mM dithiothreitol (DTT) (Sigma) at 95 °C for 5 mins, and subsequently alkylated in 50 mM iodoacetamide (IAA) (Sigma) for 30 mins in the dark at room temperature. After alkylation, the samples were transferred to a 10 kD centrifugal spin filter (Millipore) and sequentially washed with 200 mL of 8 M urea for three times and 200 mL of 50 mM ammonium bicarbonate for two times by centrifugation at 14,000 g. Next, tryptic digestion was performed by adding trypsin (Promega) at 1:50 (enzyme/substrate, m/m) in 200 mL of 50 mM ammonium bicarbonate at 37°C for 16 hrs. Peptides were recovered by transfer-ring the filter to a new collection tube and spinning at 14,000 g. To increase the yield of peptides, the filter was washed twice with 100 mL of 50 mM ammonium bicarbonate. Peptides were desalted by StageTips (34, 35).

GO and KEGG pathway analysis Gene Ontology (GO) is an ontology widely used in the field of bioinformatics for annotating large scale genes and gene products (36). KEGG is a practical database resource for genome sequencing and polymer experiment technology. It is generated by molecular- level information, especially macromolecular datasets, which can be used to predict which by pathways of a particular gene is enriched (37). It covers information resources such as diseases and pathways, GO analysis and KEGG analysis were performed by DAVID tools (https://david.ncifcrf.gov/). P <0.01 was considered statistically significant.

### Statistical Analysis

Group data are displayed as means ± standard errors of the mean (SEMs). Differences between groups were calculated by Student’s unpaired two-tailed t-tests or one-way analyses of variance (ANOVAs) followed by Tukey’s *post hoc* tests. P < 0.05 was considered statistically significant.

## Results

### SNAPIN Expression Increases With the Age of Mice and SNAPIN Is Down-Regulated in Diabetic Mice

**Figure 1:**
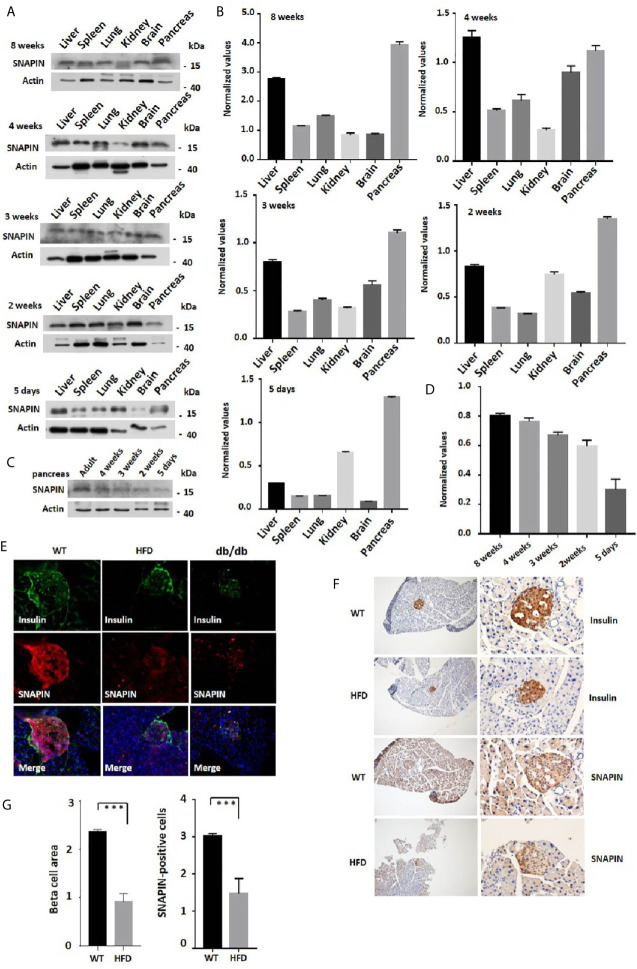
Expression of SNAPIN in mouse tissues. **(A)** SNAPIN expression was calculated by Western blot. SNAPIN was comparable for 5 days to 8 weeks mouse. **(B)** Normalized histograms of snapin protein against actin by using imaging software. **(C)** The expression level of SNAPIN in mouse pancreas also closely increased with the age of mice. **(D)** Normalized histograms of snapin protein against actin by using imaging software. **(E)** Representative immunofluorescence images of mouse healthy and pre-diabetic pancreases using antibodies against SNAPIN and insulin. The insulin and SNAPIN of HFD mice and db/db mice were significantly lower than those of wildtype mice. **(F)** Pancreatic β cell size and SNAPIN expression were calculated from the immunohistochemistry (IHC) sections processed for insulin and SNAPIN by capturing bright field images (Leica CTR 4000 series). The β cell size of high-fat diet (HFD) mice were significantly smaller than that of wildtype mice, and SNAPIN expression of HFD mice were significantly lower than that of wildtype mice. Scale bar: 100µm (left) and 50 µm (right). Values are expressed as means ± S.E.M. P<0.001 versus control. **(G)** Statistics for β cell size and SNAPIN expression. ***P < 0.001.

Tissues of six mice of different ages were isolated, followed by protein extraction to detect the expression of SNAPIN (**Figures 1A, B** **)**. We found that the expression of SNAPIN was relatively higher in liver (38), kidney (39) and pancreas (25). The expression level of SNAPIN in mouse pancreas also closely increased with the age of mice (**Figures 1C, D** **)**.

We next investigate whether SNAPIN expression has discrepancy in its involvement with healthy and diabetic mice. Hematoxylin staining was performed to detect insulin and SNAPIN expression in wild-type and diabetic mice. Decreased β cell masses and decreased SNAPIN were observed in diabetic mice compared with WT controls. Diabetic mice had much less SNAPIN compared with WT controls (**Figure 1E**), tending to a 40% reduction. We also investigated SNAPIN expression by immunostaining, and diabetes mice had much less SNAPIN compared with WT controls (**Figures 1F, G** **)**.

### SNAPIN Is Mostly Located in the Cytoplasm, and Is Co-Located With Insulin Secretory Granules

**Figure 2:**
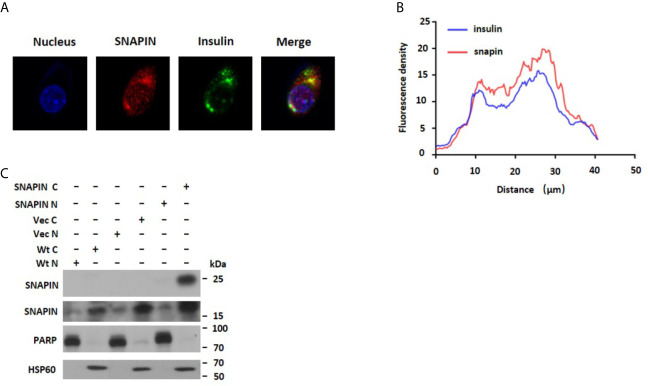
Subcellular localization of SNAPIN. **(A)**. Immunofluorescence was performed to measure the subcellular localization of SNAPIN. SNAPIN was visualized in INS-1 and Min6 cells using anti- SNAPIN antibody in conjunction with anti-rabbit FITC, whereas insulin was visualized using anti-insulin and red-conjugated goat anti-mouse. Slides were mounted and fluorescence viewed under a laser scanning confocal microscope equipped with a 488 nm laser excitation filter for FITC. The nuclei were labeled with DAPI (blue) (4ʹ,6-diamidino-2-phenylindole), and SNAPIN protein was labeled with the Alesa Fluor 643 (red). Scale bar, 2 mm. **(B)** The fluorescence distribution of SNAPIN and insulin was analyzed by ImageJ. **(C)** Nucleocytoplasmic separation was performed to measure the Subcellular localization of SNAPIN. PARP is the nuclear marker, and HSP60 is the cytoplasmic marker. SNAPIN is mainly located in the cytoplasm.

SNAPIN was originally reported in the neurons and exclusively on the synaptic vesicle membrane (40). Later, extrarenal expression has been reported in several tissues and cell lines, including pancreatic β cells, INS1 cells, Min6 cells, brain, etc. In 3T3-L1 adipocytes, biochemical and immunofluorescence microscopy analysis showed that it locates in both diffuse cytosolic and perinuclear membrane compartments (41). We found that SNAPIN was mostly located in the cytoplasm, and we found that SNAPIN presents some diffuse cytosolic staining and is clustered into punctate structures within the cell body of in rat INS1 cells and Min6 cells. Furthermore, the punctate staining pattern was similar to that seen for insulin-containing secretory granules. The two fields were then superimposed (**Figure 2A**). And fluorescence co-localization analysis showed that SNAPIN and insulin had a co-localization relationship by ImageJ (**Figure 2B**). Data analysis confirmed that there was significant co-fluorescence between the punctate insulin and SNAPIN signals, which indicates the co-localization of SNAPIN and insulin secretory granules. We also use a wildtype Min6 cell to detect the subcellular localization of SNAPIN and found that nucleocytoplasmic separation showed that SNAPIN was predominantly located in cytoplasm (**Figure 2C**).

### SNAPIN and Its Interacting Protein- Enriched Cell Cycle

**Figure 3:**
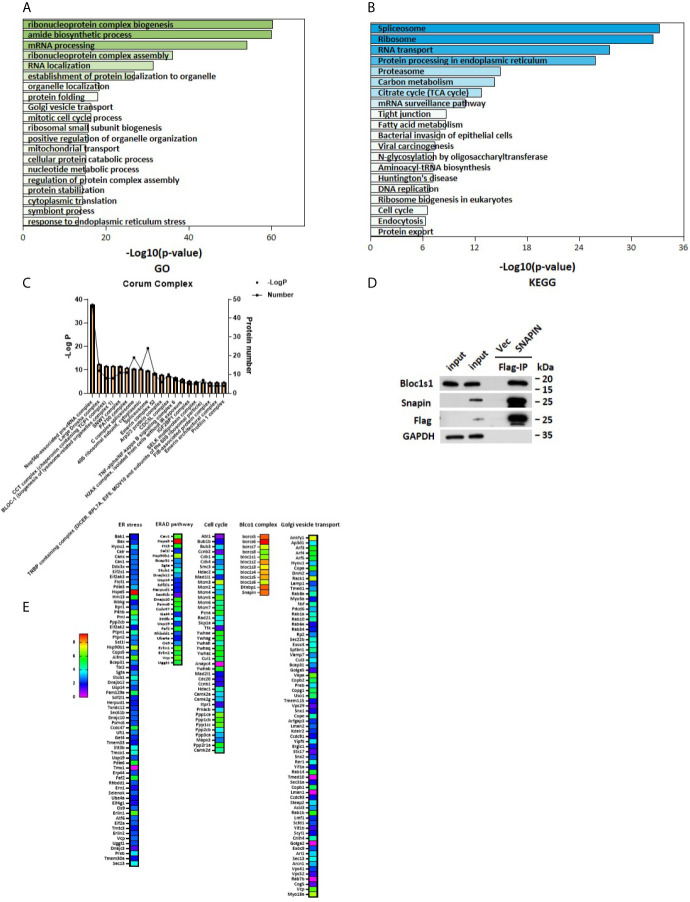
Outputs of proteomic analysis for SNAPIN -altered proteins. Identity of the SNAPIN -binding proteins. Experimental workflow of the identification of the SNAPIN -interacting proteins. Min6 cells were transduced with SNAPIN with specific sequences containing the 3xFlag tag. The resulting cell lysate was reacted with flag beads. The bound fraction was eluted and submitted for shotgun LC–MS/MS. **(A, B)** Pathways were enriched for common genes by employing the Database for Annotation, Visualization and Integrated Discovery (DAVID) ver. 6.8, KEGG pathway and GO pathway that represent functional classes of SNAPIN interacting proteins. **(C)** Proteins significantly interacting with SNAPIN were classified into different protein complexes. **(D)** IP was performed to measure the efficiency of Flag pulldown. BLOCS1 was the positive control. **(E)** The SNAPIN - binding proteins were identified by Flag Immunoprecipitation pulldown. The heat map shows the score sequest HT, where the intensities in blue indicate the relative abundance of the protein in the sample.

SNAPIN is mostly located in the cytoplasm, and to elucidate the role of SNAPIN in the development and progression of diabetes, a schematic outline of the immunoprecipitation mass spectrometry procedure was used in this study. Flag- SNAPIN or empty vector was overexpressed in Min6 cells and immunoprecipitated using whole cell lysates in triplicates. Min6 cells expressing empty vector alone served as negative controls. To identify putative biological processes associated with SNAPIN interacting proteins, we performed enrichment analysis in the Gene Ontology (GO) domain “Biological Process” (**Figure 3A**) and KEGG pathway (**Figure 3B**). This analysis identified cell cycle which related to cell growth.

Immunoblot analysis of the immunoprecipitated fractions showed that tagged- SNAPIN was enriched in the pulldown(**Figure 3C**). In contrast, no SNAPIN was detected in the pulldown in the vector control. Some papers have previously demonstrated that SNAPIN associates with Bloc1s1 by immunoblot (42). As expected, Bloc1s1 was detected in the current IP-MS study (**Figure 3C**). These data demonstrate that tagged- SNAPIN can be efficiently and specifically immunoprecipitated from cell extracts.

Proteins significantly interacting with SNAPIN were classified into different protein complexes. The results of this analysis identified twenty predominant complexes:(1) Nop56p-associated pre-rRNA complex, (2) Large Drosha complex, (3) CCT complex (chaperonin containing TCP1 complex), (4) SNW1 complex, (5) PA700 complex, (6) C complex spliceosome, (7) CDC5L complex, (8) TNF-alpha/NF-Kappa B signaling complex and other known complexes: BLOC1 (biogenesis of lysosome-related organelles complex1) complex and Arp2/3 protein complex (**Figure 3D**).

Our experiments showed that SNAPIN could affect cell proliferation and cell cycle, and we analyzed the cell cycle pathway, which are closely related to cell growth (**Figure 3E**).

### SNAPIN Overexpression Induces Proliferation of β Cells

**Figure 4:**
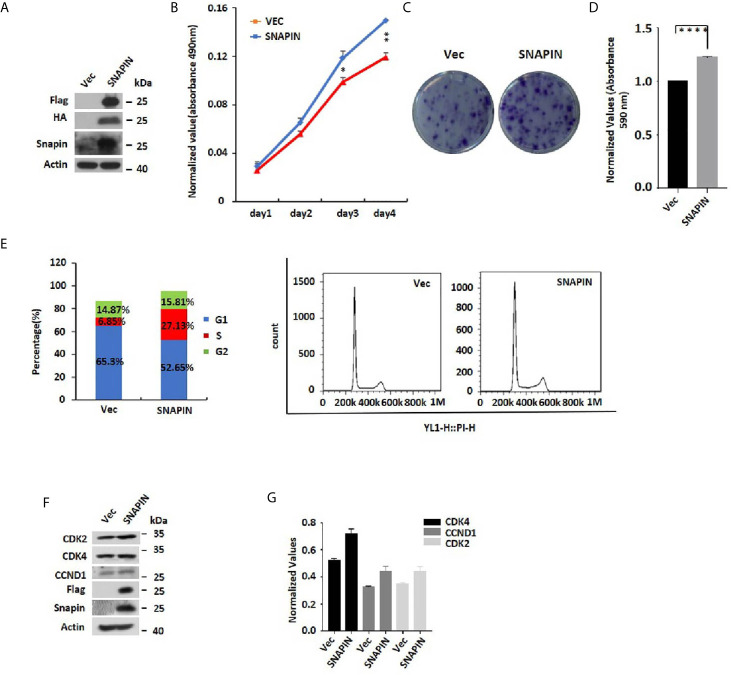
SNAPIN overexpression induces proliferation of β cells. Min6 cells were transduced with non-specific and SNAPIN specific Sequences. **(A)** Min6 cells were treated with lentivirus. A significant increase in SNAPIN protein by Western blot analysis. **(B)** MTS analysis illustrated the proliferation of Min6 cells induced after SNAPIN overexpression. **(C, D)** Clone formation showed that SNAPIN overexpression induced the proliferation of β cells. **(E)** The cells were stained with PI staining. Adherent cells were collected, and cell cycle analysis was done by flow cytometry. SNAPIN overexpression increased the population of cells in the S phase. **(F)** The protein expression levels of CyclinD1, CDK2 and CDK4 were checked by Western blot with indicated antibodies. **(G)** Normalized histograms of snapin protein against actin by using imaging software. *P < 0.05, **P < 0.01, ****P < 0.0001.

SNAPIN and its interacting proteins may enrich the cell cycle pathway, which could lead to increased cellular proliferation. To directly evaluate the role of SNAPIN in β cell proliferation, we transfected Min6 cells with lentivirus for 24 hrs. MTS analysis was applied to detect the cell proliferation. We observed a significant increase in SNAPIN protein abundance in lentivirus-treated Min6 cells by Western blot analysis (**Figure 4A**). SNAPIN overexpression induced β cell proliferation (**Figure 4B**) and increased Min6 cells clone formation compared to that control group (**Figure 4C**). **Figure 4D** shows the clone statistics.

Our MS data showed an enrichment in SNAPIN and its interacting proteins that are known to regulate the cell cycle. To provide direct evidence that SNAPIN regulates the cell cycle, we transfected Min6 cells with lentivirus to quantified cellular DNA content by flow cytometry (**Figure 4E**). Compared to the control group, SNAPIN overexpression resulted in an increase in the population of cells in the S phase from 6.85% to 27.13%. We evaluated β cells for expression of CDK2, CDK4 and CCND1, which mark the S phase of the cell cycle. CDK2, CDK4 and CCND1 expressions were all upregulated (**Figures 4F, G** **)**. Our results suggested that SNAPIN overexpression could promote the cell proliferation of β cells.

### SNAPIN Knockdown Inhibit Proliferation of β Cells

**Figure 5:**
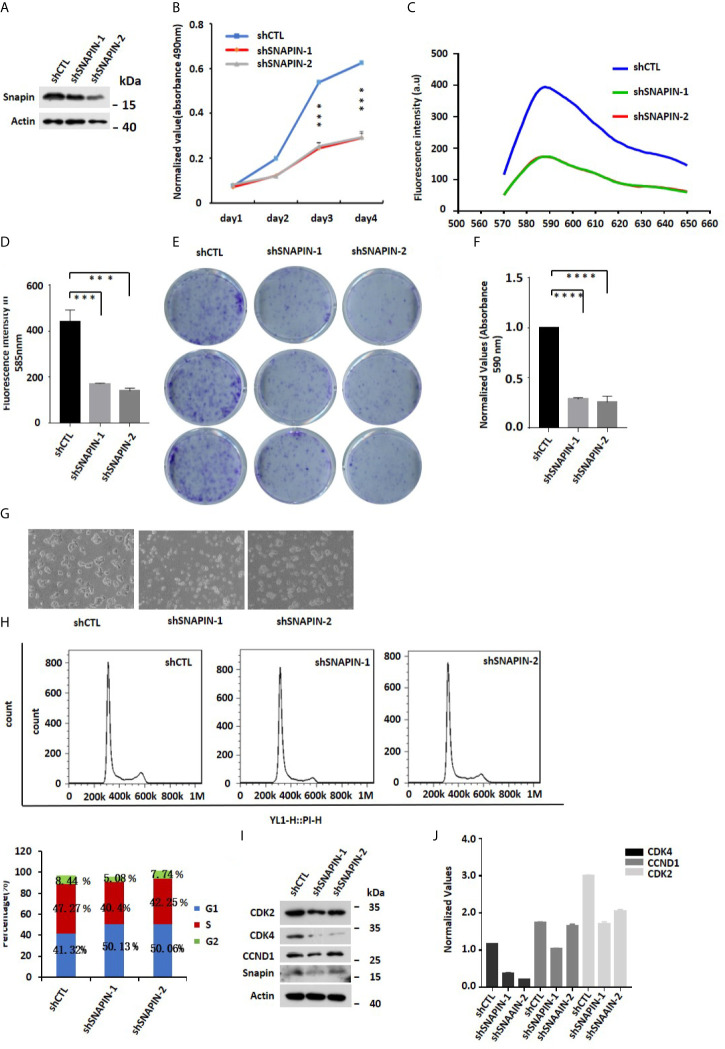
SNAPIN knockdown inhibit proliferation of β cells. **(A)** Mouse Min6 cells were transduced with lentivirus expressing shRNA and non-specific sequences. A significant decrease in SNAPIN protein by Western blot analysis. **(B)** MTS analysis illustrated that the proliferation of Min6 cells were inhibited after SNAPIN knockdown. **(C)** Click-iT™ EdU Proliferation Assay illustrated that the proliferation of Min6 cells were inhibited after SNAPIN knockdown. **(D)** Statistic for fluorescence intensity in 585nm. **(E)** Clone formation showed that SNAPIN knockdown inhibited the proliferation of β cells. **(F)** Statistic for clone formation. **(G)** Cell photography indicates Min6 cells suffer death with SNAPIN knockdown. **(H)** The cells were stained with PI staining. Adherent cells were collected, and cell cycle analysis was done by flow cytometry. SNAPIN knockdown resulted in a decrease in the population of cells in S phase. **(I)** The protein expression levels of CyclinD1, CDK2 and CDK4 were checked by Western blot with indicated antibodies. **(J)** Normalized histograms of snapin protein against actin by using imaging software. *P < 0.05, **P < 0.01, *** P< 0.001, ****P < 0.0001.

Lentivirus shRNA to silence SNAPIN and transfected Min6 cells with lentivirus for 24 hrs. MTS analysis was applied to detect the cell variety. We observed a significant decrease in SNAPIN protein abundance in lentivirus-treated Min6 cells by Western blot analysis (**Figure 5A**). SNAPIN knockdown inhibited β cells proliferation (**Figure 5B** **)** and inhibited Min6 cells clone formation compared to that of the control group (**Figure 5E**). **Figure 5F** shows the clone statistics. Cell photographic also showed that SNAPIN knockdown could cause Min6 cell death (**Figure 5G**).

We also quantified cellular DNA content by flow cytometry (**Figure 5H** **)**. Compared to control group, SNAPIN knockdown resulted in a decrease in the population of cells in the S phase from 47% to 40% and 42.25%. We evaluated β cells for expression of CDK2, CDK4 and CCND1, which marks the G1/S phase of the cell cycle. CDK2, CDK4 and CCND1 expressions were downregulated (**Figures 5I, J** **)**. Click-iT™ EdU Proliferation Assay measure β cells proliferation (**Figure 5C**). statistics for fluorescence intensity in 585 nm (**Figure 5D**). Our results suggested that SNAPIN knockdown could inhibit the proliferation of β cells.

### SNAPIN Knockdown Induce β Cells Apoptosis

**Figure 6:**
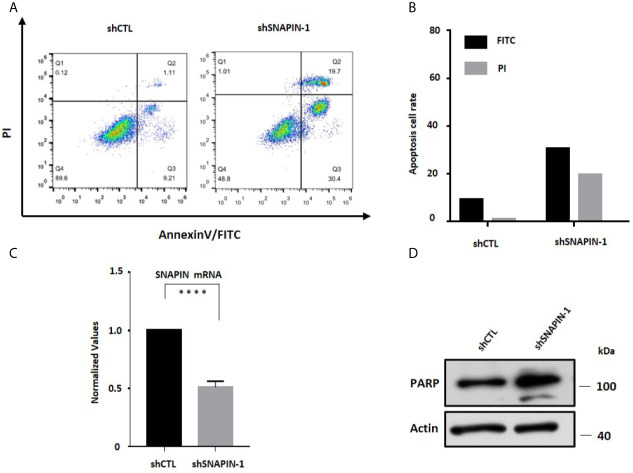
SNAPIN knockdown induce apoptosis of β cells. **(A)** apoptosis assay indicate that the apoptosis of β cells were induced after SNAPIN knockdown. **(B)** statistic for FITC and PI of apoptotic β cells. **(C)** Mouse Min6 cells were transduced with lentivirus expressing shRNA and non-specific sequences. A significant decrease in SNAPIN expression. **(D)** SNAPIN knockdown induced PARP cleavage of apoptotic marker protein. ****P < 0.0001.

Apoptosis is a specific mode of programmed cell death (43). Inappropriate apoptosis can cause a variety of diseases, so the regulation of apoptosis has great therapeutic potential (44). As a substrate of Caspase 3, PARP cleaves during the initiation of apoptosis program (45). So Cleaved PARP is a signature protein for apoptosis. In order to study the effect of SNAPIN on apoptosis of β cells, we used shRNA to interfere with SNAPIN expression and detected cell apoptosis based on Annexin V-FITC/PI staining. The proportion of apoptotic cells in the Control group was 10.32%, and the proportion of apoptotic cells after SNAPIN interference was 50.1% (**Figures 6A, B** **)**. This result indicated that interference with SNAPIN expression induced apoptosis of cells. we also analyzed the expression level of PARP and SNAPIN (**Figures 6C, D** **)**.

### SNAPIN Knockdown Inhibit Insulin Protein/mRNA Level

**Figure 7:**
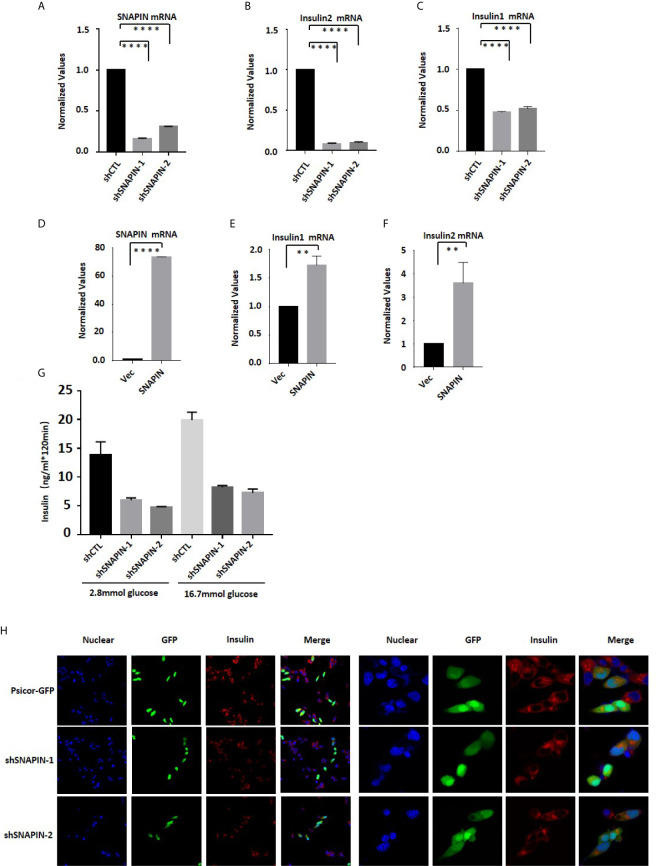
SNAPIN affects insulin mRNA and protein levels. **(A)** SNAPIN knockdown efficiency in β cells. **(B, C)** SNAPIN knockdown inhibit Insulin mRNA expression in β cells. **(D)** SNAPIN overexpression efficiency in β cells. **(E, F)** SNAPIN overexpression increased Insulin mRNA expression in β cells. QPCR was performed to measure the mRNA expression of insulin. Data are shown as the mean ± SD, n = 3. **(G)** SNAPIN knockdown inhibits insulin protein expression in INS1 cells. Glucose-induced insulin secretion measured by ELISA. **(H)** SNAPIN knockdown inhibits insulin protein expression in Min6 cells. INS1 cells were transduced with lentivirus expressing shRNA which obtained GFP. The vector was Psicor-GFP. The insulin protein expression levels were measured by immunofluorescence. The nuclei were labeled with DAPI (blue) (4ʹ,6-diamidino-2-phenylindole), insulin protein was labeled with the Alesa Fluor 643 (red) and shSNAPIN was labeled with the GFP (yellowish-green). **P < 0.01, ****P < 0.0001.

In order to evaluate the effect of SNAPIN on insulin mRNA level, we used shRNA to silence SNAPIN and transfected Min6 cells with lentivirus for 24 hrs. We observed a significant decrease in SNAPIN mRNA abundance in lentivirus treated Min6 cells by QPCR analysis (**Figure 7A**). Insulin- obtained INS1 and INS2 were inhibited after SNAPIN knockdown (**Figures 7B, C** **)**. We also transfected Min6 cells with lentivirus to quantify insulin mRNA level. We observed an increase in SNAPIN mRNA abundance in lentivirus treated Min6 cells by QPCR analysis (**Figure 7D**), and insulin only showed a slight increase (**Figures 7E, F** **)**.

Min6 cells were treated with two shRNA to interfere SNAPIN. Glucose-induced insulin secretion was measured by ELISA. Min6 cells were stimulated with 2.8mmol/L and 16.7mmol/L glucose solutions for 2 hr, and the supernatant was collected to detect insulin content. After SNAPIN interference, insulin expression was reduced (**Figure 7G**). Then, we treated INS1 cells with two shRNA to interfere SNAPIN and detected insulin by immunofluorescence. SNAPIN was visualized by GFP, and insulin was visualized using anti-insulin antibody in conjunction with goat anti-mouse cy3. Insulin secretion decreased when SNAPIN was knocked down (**Figure 7H**).

## Discussion

Diabetes mellitus is a very common metabolic disease, its acute and chronic complications have serious impacts on human health (46). Pancreatic β cells are the only cells that can produce insulin in the human body. Once the β cells are dysfunctional, the body cannot produce enough insulin to maintain the stability of blood glucose, and long-term hyperglycemia of the body will cause multiple organ dysfunction (47). The replication rate of β cells is very low, and scientists have attempted to induce the differentiation of β cells from progenitor cells or stem cells or from other islet cell lines, but the clinical progress is still very difficult. Many scientists are trying to identify small molecules or biomacromolecules that can induce β cells to self-replicate. However, there is no effective drug that can effectively induce β cells to replicate. Therefore, it is significant to find new therapeutic targets and new therapeutic methods for diabetes.

SNAPIN was originally reported to be a SNAP25-related protein that mainly interacts with SNARE complexes for vesicle transport to regulate insulin secretion (29). We compared the amino acid sequences of SNAPIN in humans, mice, and rats, and SNAPIN is highly conserved and homologous, suggesting that SNAPIN performs highly consistent biological functions in humans, mice and rats. We extracted various tissues of mice at different ages. SNAPIN was abundant in the pancreas, and its expression increases with the aging of mice, which may indicate that SNAPIN is related to the development of the pancreas. However, more experiments are needed to confirm this, we can extract the islets of mice of different gestational ages to interfere with or overexpress SNAPIN to explore the function of SNAPIN in the development of islets. SNAPIN is significantly correlated with the TMEM27 gene, which encodes a membrane protein that is cleaved and shed by pancreatic β cells, and this protein has been proposed as a biomarker for β cell quality (33). This suggests SNAPIN may also be a biomarker for β cells.

Furthermore, we used SNAPIN as bait to find SNAPIN interacting proteins and various protein complexes were enriched, including BLOC1, BORC, Arp2/3, Dynactin Complex.Snap23, Snap47 and other SNAP family members reported in the paper that interact with SNAPIN. We verify our results by using Bloc1s1 as a positive control. The proteins with high ratings were analyzed by Metascope and further enriched by BO/KEGG pathway. We enriched BLOC1/BORC, which is a SNAPIN family member, and cell cycle and protein processing in the endoplasmic reticulum etc, can further explore the mechanism. According to KEGG enrichment results, we detected changes in major proteins in the UPR pathway. Our experiment results show that SNAPIN affects the Min6 cell cycle and the level of cell cycle markers. We will continue to explore whether SNAPIN regulates β cell proliferation by interacting with cell cycle-related protein in follow-up work.

As the only glucose-lowering hormone in the body, insulin can also promote body growth (48). Our study shows that SNAPIN can promote insulin secretion, which is consistent with other reports (25). We found that SNAPIN promotes β cell growth, and it remains to be seen whether this pro-growth effect is due to SNAPIN or due to the increase in insulin secretion.

In conclusion, we showed that overexpression of SNAPIN is sufficient to induce β cell proliferation. We can therefore try to design agonists for the SNAPIN sequence as a new treatment for diabetes. This is a huge challenge but could provide new strategies for treating diabetes. Taking a broader perspective, SNAPIN overexpression promoting cell proliferation may open a new avenue of research in the field of tissue regeneration.

Finally, it is important to emphasize that there are many treatments available in addition to b cell proliferation. For example, many of the pathways that activate proliferation also activate survival pathways, another important therapeutic objective. Further, β cell expansion might be achieved through other approaches, notably β cell redifferentiation and trans-differentiation from a- or d-cells to β cells (49, 50). Of course, creating new β cells also requires effectively addressing autoimmunity. These are all critical, fertile and active areas of β cell therapeutic research.

## Data Availability Statement

The data presented in the study are deposited in the PRIDE repository, accession number PXD023786.

## Ethics Statement

The animal study was reviewed and approved by Xiamen University Institutional Animal Care and Use Committee. Written informed consent was obtained from the owners for the participation of their animals in this study.

## Author Contributions

MJ designed and performed experiments, analyzed data and drafted the manuscript. ZK designed and performed experiments and analyzed data. WW, WL, and JC conceived and designed the experiments, analyzed and interpreted data, and revised the manuscript. All authors read and approved the manuscript. All authors contributed to the article and approved the submitted version.

## Funding

This work was supported by grants from the Natural Science Foundation of China (81471081 to WW, 91953114, 81761128015, 81861130370, 31871319 to WL), the Natural Science Foundation of Fujian Province, China (2019J01010 to WW), Xiamen Research Foundation for Science and Technology Project (3502Z20194037 to WW) and Scientific Research Foundation for Advanced Talents, Xiang’an Hospital of Xiamen University (PM201809170005 to WW).

## Conflict of Interest

The authors declare that the research was conducted in the absence of any commercial or financial relationships that could be construed as a potential conflict of interest.
